# Between-and within-socioeconomic groups temporal inequality in the uptake of malaria prevention strategies among pregnant women and under-five children in Ghana (2003–2022)

**DOI:** 10.1186/s12936-025-05512-5

**Published:** 2025-08-22

**Authors:** Denis Okova, Akim Tafadzwa Lukwa, Marian Edusei, Paidamoyo Bodzo, Christian Atta-Obeng, Plaxcedes Chiwire, Charles Hongoro

**Affiliations:** 1 Health Economics Unit, School of Public Health and Family Medicine, Faculty of Health Sciences, University of Cape Town, Cape Town, South Africa; 2 Division of Family Medicine, Family, Community and Emergency Care (FaCE), Faculty of Health Sciences, University of Cape Town, Cape Town, South Africa; 3 Policy Planning Monitoring and Evaluation, The Ghana Health Service, Accra, Ghana; 4 National Malaria Elimination Programme (NMEP), Accra, Ghana; 5 Western Cape Department: Health, P.O. Box 2060, Cape Town, 8000 Western Cape South Africa; 6 Department of Health Services Research, CAPHRI Care and Public Health Research Institute, Maastricht University, 6200 MD Maastricht, The Netherlands; 7 Developmental, Capable and Ethical State, Human Sciences Research Council, Pretoria, 0001 Gauteng South Africa; 8 School of Health Systems and Public Health (SHSPH), Faculty of Health Sciences, University of Pretoria, Pretoria, 0028 Gauteng South Africa

**Keywords:** Insecticide-treated nets (ITNs), Intermittent preventive treatment in pregnancy (IPTp), Socioeconomic inequalities, Erreygers normalized concentration index (ENCI), Theil index

## Abstract

**Background:**

Malaria remains a leading cause of morbidity and mortality among pregnant women and children under five in sub-Saharan Africa. Despite over two decades of efforts including insecticide-treated net (ITN) distribution and intermittent preventive treatment in pregnancy (IPTp), universal and equitable coverage has not been achieved. In Ghana, coverage disparities persist, particularly along socioeconomic and geographic lines. This study investigates temporal trends and decomposes both within-group and between-group socioeconomic inequalities in ITN use and IPTp coverage among children and pregnant women in Ghana.

**Methods:**

This study analysed nationally representative data from the Ghana Demographic and Health Surveys (2003, 2008, 2014, and 2022), focusing on ITN use among pregnant women and children under five, and IPTp uptake. Inequality was assessed using the Erreygers normalized concentration index (ENCI) and the Theil index (GE 2). Theil indices were then decomposed to quantify within- and between-group contributions by socioeconomic status (SES) and residence (urban versus. rural).

**Results:**

ITN use among under-five children increased modestly from 58.1% (2003) to 62.9% (2022); among pregnant women, usage fluctuated but returned to 60% in 2022. IPTp coverage rose markedly from 0.85% (2003) to 60% (2022). ENCI values showed that ITN use was consistently pro-poor, becoming more concentrated among the poor over time (e.g., ENCI for pregnant women: − 0.04 in 2003 to − 0.32 in 2022). In contrast, IPTp coverage shifted to a pro-rich distribution from 2008 onwards. Theil decomposition revealed that most observed inequalities were driven by within-group disparities (e.g., within SES or residence categories), though between-group inequality increased over time particularly for IPTp. For example, in 2022, 85% of ITN inequality among pregnant women was within SES groups, while 18% was attributable to between-group differences.

**Conclusion:**

Despite overall improvements in malaria prevention uptake, substantial socioeconomic inequalities persist especially within social and geographic subgroups. Equity-focused strategies must complement national-level efforts by addressing barriers specific to underserved populations, including informal urban settlements and remote rural communities. Policymakers must prioritize targeted, locally responsive interventions to reduce both within- and between-group disparities and achieve Ghana’s malaria elimination and health equity goals.

## Background

Malaria is one of the leading causes of mortality among pregnant women and children in Africa [1]. In 2021, the World Health Organization (WHO) reported approximately 593,000 deaths and 234 million malaria cases in Africa, with sub-Saharan Africa (SSA accounting for 90% of these cases [1, 2]. Cost-effective interventions used to reduce malaria in the region since the launch of the Roll Back Malaria (RBM) programme in 1998 include insecticide-treated nets (ITNs) and intermittent preventive treatment for pregnant women (IPTp) [3]. The WHO recommends that 80% of the high-risk populations specifically pregnant women and under five children should have access to and utilize malaria prevention interventions, particularly ITNs and IPTp, to achieve universal protection [4]. Between 2004 and 2020, 2.3 billion insecticide-treated mosquito nets (ITNs) were supplied globally, with 229 million nets distributed to malaria-endemic regions [5, 6]. SSA received about 91% of these interventions. Despite these efforts, universal coverage has not been achieved, with only 65% of households in sub-Saharan Africa owning at least one insecticide-treated net (ITN) as of 2020 [7].

In Ghana, similar concerns regarding ITN and IPTp use among children and pregnant women have been identified [8, 9]. Despite the Ghana National Malaria Control Programme’s (NMCP) efforts to distribute ITNs in clinics, schools, and community centres [8], full coverage of these preventive interventions has not been achieved [10]. As of 2021, Ghana had achieved only 47% coverage of insecticide-treated nets (ITNs), defined as the proportion of the population with access to at least one ITN per two people in the household [11, 12]. This statistic, which reflects ITN ownership rather than actual usage, is based on data from the Demographic and Health Survey (DHS) [13]. Studies by Klu et al*.* [8] and Kanmiki et al. [9] in Ghana found that although ITN ownership was higher among wealthier households, usage among pregnant women and children was paradoxically lower among the richest quintiles. For example, Kanmiki et al. [9] reported that approximately 74% of total ITN ownership was concentrated in the wealthiest segments of the population (as measured by concentration indices), yet the richest were 33% less likely to use ITNs and IPTp compared to the poorest quintile. Additionally, Kanmiki et al*.* [9] identified inequalities in ITN ownership and utilization between rural and urban populations, with ownership being 74% concentrated among the richest, while the richest were 33% less likely to use ITNs and IPTp [9].

Budu et al*.* [14] revealed that poor pregnant women were more likely to receive IPTp treatment compared to wealthy women, although IPTp coverage was relatively higher among educated women. Socioeconomic inequalities in the use and ownership of ITNs and IPTp remain a critical issue to be addressed if Ghana aims to achieve its sustainable development goals and eradicate malaria by 2030. Between-group inequalities refer to disparities between different socioeconomic groups while within-group inequalities examine disparities within each socioeconomic group regarding ITN and IPTp coverage [15]. In Ghana, previous studies have focused on socioeconomic factors and risk associations related to ITN and IPTp coverage but have not comprehensively assessed within- and between-group inequalities among pregnant women and children [4, 8].

In Ghana, despite significant efforts to distribute and promote the use of ITN and IPTp, stark socioeconomic inequalities in their utilization persist [14]. These inequalities are evident both between different socioeconomic groups and within each group. Studies such as those by Klu et al*.* and Kanmiki et al*.* have highlighted a disparity in ITN and IPTp usage, with wealthier households owning more ITNs, but utilizing them less frequently compared to poorer households [8, 9]. Additionally, Kanmiki et al*.* revealed that ITN ownership is disproportionately higher among the wealthiest, yet these individuals are less likely to use them effectively [9]. Conversely, Budu et al*.* found that ITN use among children under five and IPTp receipt among pregnant women is pro-poor, indicating higher usage among less affluent populations [14]. A multicountry study that included Ghana found widespread socioeconomic inequalities in malaria intervention coverage, with poorer populations consistently experiencing lower access [16]. Most studies on malaria prevention inequalities among pregnant women and children under five focus on broad between-group differences, such as by wealth or urban–rural residence. However, this often overlooks critical within-group disparities, such as variations tied to remoteness in rural areas or informal housing in urban settings. Ignoring this internal heterogeneity can obscure hidden vulnerabilities and undermine the effectiveness of targeted public health interventions [17]. Addressing both forms of inequality is essential; between-group evidence informs group-based targeting, while within-group insights guide more localized and tailored policy responses.

This study addresses the often-overlooked issue of within-group disparities in malaria prevention by examining both between- and within-group socioeconomic inequalities in ITN and IPTp coverage among children under five and pregnant women in Ghana. Drawing on four rounds of DHS data (2003, 2008, 2014, and 2022), it analyses temporal trends and decomposes the contributions of residence (rural vs. urban) and wealth to these disparities. The findings offer actionable insights for policy by identifying subpopulations, such as rural communities with poor service coverage and urban informal settlements, that may be missed by broad national strategies. By highlighting where and how inequalities persist, the study supports the design of more precise, equity-focused malaria interventions aligned with Ghana’s malaria elimination and universal health coverage goals.

## Methods

### Data

This study utilized nationally representative data from the Ghana Demographic Health Surveys for the years 2003 [18], 2008 [19], 2014 [20], and 2022 [13]. These publicly available datasets included population samples as follows: 5,691 women and 3,844 children under five years old in 2003; 4,916 women and 2,992 children under five years old in 2008; 9,396 women and 5,884 children under five years old in 2014; and 15,014 women and 9,353 children under five years old in 2022.

### Outcome variables

This study assessed three malaria prevention strategies: ITN use by pregnant women, ITN use by children under five, and IPTp coverage. ITN use by pregnant women was defined as women aged 15–49 who, in a household with at least one ITN, slept under an ITN the night before the survey [21]. Similarly, ITN use by children under five was defined as those who, in a household with at least one ITN, slept under an ITN the night before the survey [22]. IPTp coverage was defined as women aged 15–49 who had a live birth or stillbirth in the two years preceding the survey and received three or more doses of sulfadoxine-pyrimethamine (SP), also known as Fansidar, during their pregnancy [23].

### Socioeconomic and living conditions

Socioeconomic status (SES) in this study was measured using the standard DHS wealth index, which is derived through principal component analysis (PCA) of household asset ownership and housing characteristics. The present study relied on the DHS-calculated wealth index, without recalculating the PCA [24]. The original index classifies households into five quintiles: poorest, poorer, middle, richer, and richest. However, for the purposes of this study, these five groups were categorized into three broader SES categories: poor (combining poorest and poorer), middle, and rich (combining richer and richest). This re-categorization was necessary for two main methodological reasons. First, several of the original quintiles had relatively small numbers of observations, particularly within key analytic strata such as geographic regions and sex-specific subgroups. This posed a risk of model instability and inflated standard errors in regression analyses. Second, the re-grouping facilitated the decomposition of inequality using Theil indices. The Theil decomposition requires meaningful group stratification, and sparse subgroups can distort the partitioning of within- and between-group components [25, 26]. Consolidating into three categories ensured more balanced group sizes and clearer interpretability of inequality measures.

Consolidating into three categories helped ensure meaningful group sizes and clearer interpretation of SES-related disparities. This approach has precedent in prior research, including a study by Mare et al. [27], which consolidated DHS quintiles into three categories to improve the validity and interpretability of multivariable models. Residence status was operationalized as a binary variable, with urban living coded as 1 and rural living coded as 0. For each survey year, the variables were generated and recoded as necessary. Subsequently, the datasets were appended to create a comprehensive panel dataset, facilitating longitudinal analysis across the survey years.

### Statistical analysis

All analyses for this study were conducted using STATA version 15 (Stata Corp. Inc., College Station, TX, USA). All analyses accounted for DHS sampling weights and survey design.

### Descriptive statistics

Descriptive statistics were computed as weighted proportions with 95% confidence intervals to summarize the prevalence of each outcome across four survey years. In addition, pairwise comparisons using design-based Pearson chi-square tests were conducted between adjacent survey years (2003 vs. 2008, 2008 vs. 2014, and 2014 vs. 2022) to test for statistical significance in outcome prevalence changes. All statistical significance was assessed at a 5% level, with p-values below 0.05 considered significant.

### Erreygers normalized concentration index

To assess socioeconomic inequalities in insecticide-treated net (ITN) usage and IPTp coverage among women and children, this study employed the Erreygers Normalized Concentration Index (ENCI) [28–30]. The ENCI is particularly suited for binary health outcomes, such as ITN use and IPTp uptake, because it adjusts for the bounded nature of such variables and ensures that the index satisfies key properties of inequality measurement, including mirror (reversing the outcome flips the sign of the index but retains the magnitude) [28], transfer (a transfer of a small amount of heath service/benefit from a richer to a poorer individual reduces inequality) [30], and level independence (inequality remains unchanged if all individuals experience the same absolute gain) [29]. The standard concentration index, while widely used, is problematic for binary variables because its bounds depend on the mean of the health variable, leading to inconsistencies when comparing across populations or over time [28, 31]. The Wagstaff correction [32], though it addresses part of this issue, does not fully satisfy the mirror property i.e., it does not produce symmetric inequality values when measuring attainment versus shortfall.

The ENCI, in contrast, provides a normatively consistent and symmetric measure of inequality for binary outcomes [29, 30], making it preferable for equity analyses in health service coverage. Its use has been widely endorsed and applied in similar public health inequality assessments [33–35]. In this case, where key outcome variables are binary and bounded (e.g., ITN usage: yes/no), the ENCI offers an interpretable measure of socioeconomic disparity, with values ranging from −1 (entirely concentrated among the poor) to + 1 (entirely concentrated among the rich), and 0 indicating perfect equality. The Erreygers normalized concentration index is calculated using the following formula;

$$E\left(C\right)=\frac{4\mu }{b-a}C$$

where:

μ is the mean of the health variable; ITN use in pregnancy, ITN use by children under-five and IPTp coverage.

*b* and *a* are the upper and lower bounds of the health variable, respectively.

*C* is the traditional concentration index.

The traditional concentration index is calculated as shown below;

$$C=\frac{2}{\mu } \text{Cov} (y,R)$$

where:

*y* is the health variable (ITN use or IPTp uptake).

μ is the mean of *y.*

*R* is the fractional rank of individuals in the distribution of SES.

Cov denotes the covariance between *y* and *R.*

### Analytical approach

To estimate socioeconomic inequality in health outcomes, the ENCI was computed using Stata’s conindex command. The analytical procedure involved several steps. First, individuals were ranked by their SES, from poorest to richest, and each was assigned a fractional rank (*R*) based on their relative position in the wealth distribution. Second, the mean (μ) of the health variable (*y*) was computed. Third, the covariance between μ and *y* was computed. Fourth, the traditional (unnormalized) concentration index (CI) was derived as the ratio of the covariance to the mean of *y*. Finally, the ENCI was computed using Erreygers’ normalization formula which adjusted for the bounded nature of the health variables ensuring compliance with key properties of inequality measurement (mirror, transfer and independence). To quantify uncertainty, the cluster () option in the conindex command, was used specifying the primary sampling unit (PSU) variable from the DHS. This produced cluster-robust standard errors, which account for intra-cluster correlation using a Huber–White sandwich estimator [36]. The output included the standard error (SE)**,** and the p-value was calculated from a Z-statistic comparing the ENCI estimate to its standard error [34]. A p-value < 0.05 was interpreted as statistically significant, indicating evidence of inequality. SE quantifies the precision of the ENCI estimate; smaller SEs indicate more precise estimates, while larger SEs reflect greater sampling variability.

### Theil index

To further measure and decompose inequality in the use of ITNs and IPTp coverage, the Theil index particularly the Generalized Entropy index GE(2) was used. This index allows for a clear distinction between within-group and between-group components of inequality. The present study selected GE(2) because it is particularly sensitive to disparities at the upper end of the distribution [37], making it well-suited for this study’s objective of assessing whether gains in coverage disproportionately benefit individuals in higher socioeconomic strata. While GE(0) and GE(1) are also valid measures [38], with GE(0) being more sensitive to lower-tail inequality and GE(1) applying equal weight across the distribution, GE(2) aligns more directly with this study’s interest in identifying pro-rich patterns of inequality in service uptake. The GE(2) ranges from 0 to infinity, with 0 indicating perfect equality and higher values denoting greater inequality [37, 39].

### Generalized entropy index GE(2)

The GE(2) index is calculated as follows:

$$GE\left(2\right)= \frac{1}{2}{\sum }_{i-1}^{n}({\frac{{y}_{i}}{\mu })}^{2}-1$$

where;

y_i_ is the outcome for individual i,

μ is the mean of the outcome variable.

n is the total number of individuals.

### Decomposition into within-group and between-group inequality

The overall GE(2) index can be decomposed into within-group and between-group components:

$$GE\left(2\right)= {GE}_{w}\left(2\right)+ {GE}_{B}(2)$$

where;

${GE}_{w}\left(2\right)$ represents within-group inequality and.

${GE}_{B}(2)$ represents between-group inequality.

### Calculation of within-group and between-group contributions

The within-group and between-group contributions to overall inequality were calculated as follows:

$$Within-group contribution= \left(\frac{{GE}_{w}(2)}{GE(2)}\right)*100$$

$$Between-group contribution= \left(\frac{{GE}_{B}(2)}{GE(2)}\right)*100$$

For each survey year and each outcome variable (ITN usage by under-fives, ITN usage by pregnant women, and IPTp coverage), the following steps were performed:

- *Calculation of overall GE(2)*: The overall inequality in the outcome variable was measured using the GE(2) index.
- *Decomposition by socioeconomic status*: The overall GE(2) was decomposed into within-group and between-group components based on wealth quintiles [1–3;poor,middle,rich].
- *Decomposition by residence status*: GE(2) was also decomposed by residence status, operationalized as a binary variable (urban vs. rural). In this case, between-group inequality reflects differences in average uptake between urban and rural populations, while within-group inequality captures variation within each residence category. Although a binary classification may not capture the full spatial heterogeneity or nested geographic structures, it provides a clear and interpretable estimate of how much overall inequality is driven by broad geographic disparities in access. This approach is consistent with previous studies in sub-Saharan Africa that have documented urban–rural differences in malaria prevention coverage, including ITN use and IPTp uptake [14, 40–42].
- *Calculation of percentage contributions*: The contributions of within-group and between-group inequalities were calculated as described above.

## Results

### Descriptive statistics

The prevalence of ITN use among children under-five increased slightly from 58.11% in 2003 to 60.19% in 2008 to 60.49% in 2014 to 62.86% in 2022. However, none of the pairwise differences between adjacent years were statistically significant (Table 1). The prevalence of ITN use in pregnant women was 60% in 2003; this dropped in 2008 (52%) and 2014 (54%) and then rose back to 60% in 2022. Similarly, these year-on-year changes were not statistically significant. IPTp coverage on the other hand, increased steadily over time; from 0.85% in 2003 to 28.07% in 2008 to 38.97% in 2014 and then 60% in 2022 with all pairwise differences statistically significant at the p < 0.05 level **(**Table 2**)**. As shown in Table 1**,** higher proportions of ITN use among children under five was consistently reported in rural areas compared to urban areas across all the four years. In terms of socioeconomic distribution, the highest proportions of ITN use in under-fives was evidenced among the poor while the lowest proportions reported among the middle class for all the survey years.

**Table 1:** Sample distribution and proportions of ITN use among children under 5 for Ghana 2003, 2008,2014,2022

Characteristics	Proportions of ITN use among under-5 children			
	2003 n [CI]	2008 n [CI]	2014 n [CI]	2022 n [CI]
Residence status				
Urban	16.40 [10.47,24.75]	18.31 [15.92,20.98]	20.90 [18.62,23.38]	22.33 [20.51,24.26]
Rural	41.71 [33.40,50.52]	41.87 [38.70,45.11]	39.60 [36.78,42.49]	40.53 [38.11,42.99]
Socioeconomic status				
Poor	33.99 [26.24,42.71]	30.79 [27.84,33.91]	31.29 [28.35,34.40]	35.93 [33.41,38.54]
Middle	10.03 [6.17,15.90]	10.57 [8.81,12.64]	12.93 [11.28,14.78]	12.73 [11.36,14.25]
Rich	14.08 [8.92,21.51]	18.82 [16.46,21.42]	16.27 [14.10,18.71]	14.19 [12.67,15.85]
TOTAL	58.11 [50.94,64.95]	60.19 [56.95,63.33]	60.49 [58.04,62.90]	62.85 [60.59,65.06]
Pairwise Pearson chi-squared test		0.5657	0.8835	0.2152

Source: Author computations *CI* Confidence intervals

**Table 2:** Sample distribution and proportion of ITN use and IPTp coverage among pregnant women for Ghana 2003, 2008, 2014,2022

	ITN Use (Pregnant Women)				IPTp Coverage			
	2003 % [CI]	2008 % [CI]	2014 % [CI]	2022 % [CI]	2003 % [CI]	2008 % [CI]	2014 % [CI]	2022 % [CI]
Residence Status								
Urban	11.86 [5.30,77.31]	14.18 [9.88,19.94]	19.17 [15.86,22.98]	20.88 [17.67,24.50]	0.26 [0.00,0.84]	11.61 [9.47,14.17]	19.08 [16.16,22.38]	28.76 [26.65,30.96]
Rural	48.01 [10.84,87.52]	37.50 [31.47,43.94]	34.85 [30.43,39.54]	38.51 [34.78,42.39]	0.58 [0.29,1.16]	16.46 [14.23,18.95]	19.89 [17.42,22.62]	30.68 [28.38,33.08]
Socioeconomic Status								
Poor	37.22 [7.58,81.07]	26.72 [20.42,34.14]	25.70 [21.61,30.27]	33.52 [29.56,37.73]	0.26 [0.00,0.84]	10.92 [9.10,13.04]	15.75 [13.11,18.81]	24.54 [22.23,27.00]
Middle	5.20 [2.30,56.75]	8.83 [5.25,14.50]	13.92 [10.66,17.96]	12.11 [9.84,14.84]	0.11 [0.00,0.46]	4.96 [3.74,6.56]	7.14 [5.78,8.80]	12.31 [10.89,13.87]
Rich	17.45 [1.52,74.30]	16.12 [11.38,22.34]	14.40 [11.19,17.96]	13.76 [11.00,17.08]	0.47 [0.22,1.03]	12.19 [10.07,14.68]	16.07 [13.90,18.51]	22.59 [20.56,24.76]
Total	59.87 [14.10,93.13]	51.68 [44.03,59.25]	54.02 [49.31,58.65]	59.40 [54.82,63.81]	0.85 [0.47,1.53]	28.07 [25.09,31.25]	38.97 [35.73,42.30]	59.43 [56.85,61.97]
Pairwise Pearson chi-squared test		0.5340	0.6021	0.1354		0.000***	0.000***	0.000***

Source: Author computations *CI* Confidence intervals. ***Pairwise comparison between years is statistically significant (p < 0.05)

Table 2 shows the distributions and proportion of ITN use and IPTp coverage across the four years of study. Across all years, ITN use among pregnant women remained consistently higher in rural areas compared to urban areas. In 2003, rural ITN use was 48.01% (CI 10.84–87.52) versus 11.86% (CI 5.30–77.31) in urban areas. Although the urban coverage increased steadily; reaching 20.88% by 2022, rural coverage remained higher at 38.51% (CI 34.78–42.39) in the same year. By SES, the poor consistently had higher ITN use than the middle and rich groups across all years. In 2003, ITN use was 37.22% (CI 7.58–81.07) among the poor, compared to 5.20% (CI 2.30–56.75) among the middle and 17.45% (CI 1.52–74.30) among the rich. Although coverage increased slightly across all groups by 2022, the poor remained the most covered (33.52%, CI 29.56–37.73), while usage declined slightly among the rich (13.76%, CI 11.00–17.08). IPTp coverage increased markedly across all subgroups between 2003 and 2022. In urban areas, coverage rose from a negligible 0.26% (95% CI: 0.00–0.84) in 2003 to 28.76% (95% CI 26.65–30.96) in 2022. Similarly, rural areas saw an increase from 0.58% (95% CI 0.29–1.16) in 2003 to 30.68% (95% CI 28.38–33.08) in 2022. Improvements were also evident across all socioeconomic groups. Among women in the poorest tertile, IPTp coverage increased from 0.26% (95% CI 0.00–0.84) in 2003 to 24.54% (95% CI 22.23–27.00) in 2022. Middle-income women experienced a rise from 0.11% (95% CI 0.00–0.46) to 12.31% (95% CI 10.89–13.87), while the richest group improved from 0.47% (95% CI 0.22–1.03) to 22.59% (95% CI 20.56–24.76).

### Concentration indices and curves

As per Table 3, the ENCIs for ITN use in pregnancy were negative in all survey years, indicating a consistent pro-poor distribution. While the inequality in 2003 was minimal and not statistically significant (ENCI = − 0.04, p = 0.85), it became progressively more pronounced and statistically significant in subsequent years: − 0.15 in 2008 (p = 0.04), −0.23 in 2014 (p < 0.001), and − 0.32 in 2022 (p < 0.001). A similar trend was observed for ITN use among children under five years of age**.** The ENCI values were significantly negative across all four years, indicating persistent and growing pro-poor inequality. Specifically, the index was − 0.14 in 2003 (p = 0.04), − 0.16 in 2008 (p < 0.001), − 0.22 in 2014 (p < 0.001), and − 0.28 in 2022 (p < 0.001). In contrast, IPTp coverage showed a different pattern. In 2003, there was no evidence of socioeconomic inequality (ENCI = − 0.00, p = 0.75). However, in all subsequent years, the ENCI values were positive and statistically significant, indicating a shift toward pro-rich inequality. The values were 0.10 in 2008, 0.07 in 2014, and 0.11 in 2022 (all p < 0.001), suggesting that IPTp uptake increasingly favoured women from wealthier households over time.

**Table 3:** Erreygers normalised concentration indices for ITN use and IPTp coverage

Period	Erreygers normalised concentration index	Standard error	p-value
ITN use by pregnant women			
2003	− 0.04	0.19	0.85
2008	− 0.15	0.07	0.04*
2014	− 0.23	0.05	0.00***
2022	− 0.32	0.03	0.00***
ITN use by under 5 children			
2003	− 0.14	0.06	0.04*
2008	− 0.16	0.03	0.00***
2014	− 0.22	0.02	0.00***
2022	− 0.28	0.02	0.00***
IPTp coverage			
2003	− 0.00	0.01	0.75
2008	0.10	0.03	0.00***
2014	0.07	0.02	0.00***
2022	0.11	0.02	0.00***

Author computations (p < 0.05 = *, p < 0.01 = **, p < 0.001 = ***)

**Fig. 1:**
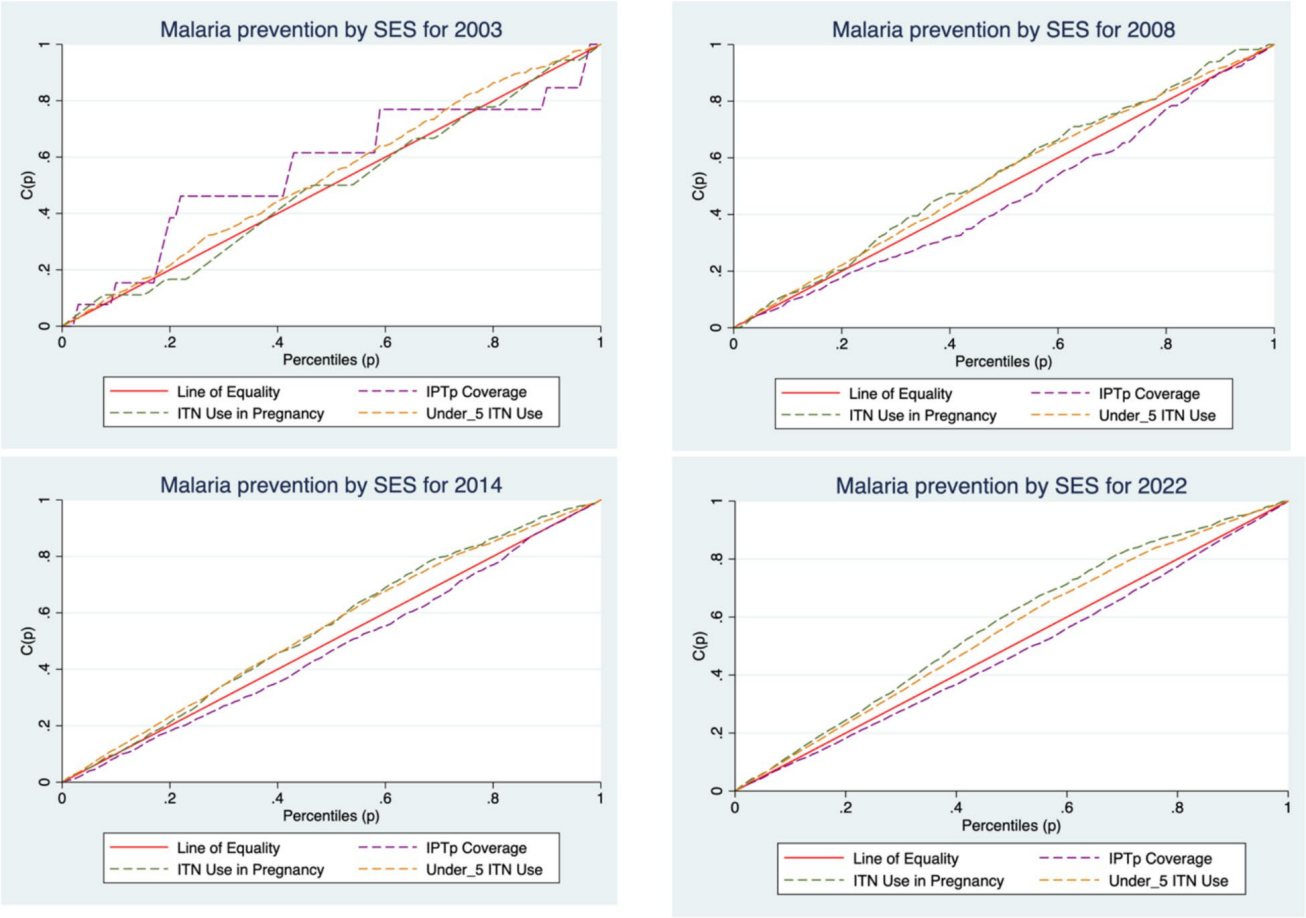
Concentration curves for ITN use in pregnancy, ITN use in under -fives and IPTp coverage for 2003,2008, 2014 and 2022

To further illustrate the pro-richness or pro-poorness in ITN use and IPTp coverage, concentrations curves shown in Fig. 1 were computed. As shown in Fig. 1**,** in 2003, IPTp coverage was pro-poor at low and middle income households and pro-rich at higher income households. For subsequent years, the concentration curves for IPTp coverage, were largely below the line of equality indicating higher IPTp coverage among rich individuals compared to poor women. With the exception of 2003, the inequalities in the three outcomes for the other three years lie clearly below or above the line of equality indicating clear pro-richness or pro-poorness.

### Decomposition analysis by residence status and SES

Table 4 presents Theil indices for ITN use among pregnant women and under-five children, decomposed by place of residence and SES, with accompanying standard errors and confidence intervals. Across all years, socioeconomic inequalities in ITN use during pregnancy are consistently higher in urban areas than in rural areas, as indicated by both higher Theil indices and statistically significant standard errors (e.g., urban 2003: Theil = 0.25, SE = 0.09, 95% CI 0.13–0.49). Moreover, these urban inequalities have widened over time rising from 0.25 in 2003 to 0.47 in 2022 while the reverse trend is observed in rural areas, where inequalities declined from 0.22 to 0.17.

**Table 4:** Theil indices for subgroups for ITN use by pregnant women and under 5 children distinguished by place of residence and SES

	Theil index decomposed by residence																							
Residence status	ITN use by pregnant women												ITN use by Under 5 children											
	2003	SE	CI	2008	SE	CI	2014	SE	CI	2022	SE	CI	2003	SE	CI	2008	SE	CI	2014	SE	CI	2022	SE	CI
Urban	0.25	0.09^***^	0.13–0.49	0.73	0.04^***^	0.38–0.52	0.58	0.02^***^	0.38–0.46	0.47	0.02^***^	0.3–0.38	0.44	0.03^***^	0.33–0.46	0.50	0.01^***^	0.35–0.40	0.49	0.01^***^	0.35–0.39	0.39	0.01^***^	0.30–0.32
Rural	0.22			0.31			0.25			0.17			0.29			0.23			0.21			0.14		

	Theil index decomposed by SES																							
SES	ITN use by pregnant women												ITN use by Under 5 children											
	2003	SE	CI	2008	SE	CI	2014	SE	CI	2022	SE	CI	2003	SE	CI	2008	SE	CI	2014	SE	CI	2022	SE	CI
Poor	0.21	0.09^***^	0.13–0.49	0.31	0.04^***^	0.38–0.52	0.26	0.02^***^	0.38–0.45	0.12	0.02^***^	0.3–0.38	0.27	0.03^***^	0.33–0.46	0.22	0.01^***^	0.35-0.0.40	0.20	0.01^***^	0.35–0.39	0.13	0.01^***^	0.30–0.32
Middle	0.25			0.39			0.22			0.28			0.17			0.39			0.28			0.25		
Rich	0.25			0.62			0.78			0.79			0.64			0.44			0.60			0.58		

Author computations; (p < 0.05 = *, p < 0.01 = **, p < 0.001 = ***)

A similar pattern is seen for ITN use among under-five children: inequalities remain consistently more pronounced in urban settings but have gradually declined over time, with Theil indices decreasing from 0.44 (SE = 0.03, CI 0.33–0.46) in 2003 to 0.39 (SE = 0.01, CI 0.30–0.32) in 2022. This declining trend, supported by narrow confidence intervals, suggests improved equity in urban ITN distribution among young children. When decomposed by SES, inequalities are highest among the rich across all years, particularly for ITN use in pregnancy, where Theil indices increased sharply from 0.25 in 2003 to 0.79 in 2014 before stabilizing at 0.64 in 2022. Among the poor, by contrast, inequalities declined from 0.21 in 2003 to 0.12 in 2022, with significant SEs and overlapping confidence intervals across years indicating a consistent reduction in disparities. Notably, for under-five children from poor households, Theil indices dropped from 0.27 to 0.13 over the same period, reinforcing the equity gains in this subgroup.

Table 5 shows the Theil index values disaggregated by residence and socioeconomic status (SES) across four survey years, alongside standard errors and confidence intervals. The findings indicate a steady contraction in socioeconomic inequalities in IPTp coverage over time across both rich and poor subgroups. Notably, the Theil index in 2003 was extremely high 68.17 in urban areas and 53.06 among the poor with very narrow confidence intervals (e.g., CI 0.99–1.00), likely reflecting extreme inequality driven by the very low IPTp prevalence that year (0.85%). The Theil index is sensitive to relative differences in a distribution. When the overall prevalence is extremely low, even small absolute differences across socioeconomic or residence groups translate into very large relative inequalities. However, as IPTp coverage expanded in subsequent years (e.g., 28.1% in 2008 and 59.4% in 2022), the Theil indices declined substantially (e.g., from 1.66 to 0.40 among the poor, and from 1.15 to 0.30 in urban areas), with consistently small standard errors (SE = 0.01) and narrow confidence intervals (e.g., CI 0.39–0.42 in 2022).

**Table 5:** Theil indices for subgroups for IPTp coverage distinguished by place of residence and SES

	Theil index decomposed by residence status											
Residence status	IPTp coverage											
	2003	SE	CI	2008	SE	CI	2014	SE	CI	2022	SE	CI
Urban	68.17	0.002^***^	0.99–1.00	1.15	0.01^***^	0.70–0.75	0.70	0.01^***^	0.59–0.63	0.30	0.01^***^	0.39–0.42
Rural	53.85			1.43			0.86			0.35		

SES	Theil index decomposed by SES											
	IPTp coverage											
	2003	SE	CI	2008	SE	CI	2014	SE	CI	2022	SE	CI
Poor	53.06	0.002^***^	0.99–1.00	1.66	0.01^***^	0.70–0.75	0.88	0.01^***^	0.59–0.63	0.40	0.01^***^	0.39–0.42
Middle	63.00			1.23			0.78			0.29		
Rich	64.17			0.96			0.64			0.24		

Source: Author computations; (p < 0.05 = *, p < 0.01 = **, p < 0.001 = ***)

Table 6 and Table 7 present the decomposition of Theil indices into within- and between-group components for ITN use (in pregnancy and among under-5 children) and IPTp coverage across four survey years. Although SEs and CIs are not repeated in these tables, they are already reported in Tables 4 and 5 for the subgroup-level Theil indices that underpin the decomposition. Therefore, the decomposed values in Tables 6 and 7 share the same SEs and CIs reported in those earlier tables. For instance, the Theil index for ITN use among pregnant women in urban areas in 2003 is 0.25 (SE = 0.09, 95% CI 0.13–0.49) as reported in Table 4. This value contributes to the overall Theil index of 0.22 in Table 6 for that year, which is entirely explained by within-group inequality (100%) indicating that the statistical uncertainty around this estimate is already captured. Similarly, the rural estimate in 2003 is 0.22 with no reported SE (likely due to smaller variance), contributing to the overall estimate.

**Table 6:** Decomposition of the Theil indices by residence status for ITN usage and IPTp coverage

	Overall within and between group inequalities by residence status							
	ITN use in pregnancy							
	2003		2008		2014		2022	
	Theil index	Contribution	Theil index	Contribution	Theil index	Contribution	Theil index	Contribution
**Overall**	0.22		0.40		0.36		0.26	
**Within-group**	0.22	100%	0.39	97.5%	0.34	94.44%	0.24	93.3%
**Between-group**	0.00		0.02	0.05%	0.02	0.06%	0.02	7.7%

	ITN use by Under 5 children							
	2003		2008		2014		2022	
	Theil index	Contribution	Theil index	Contribution	Theil index	Contribution	Theil index	Contribution
Overall	0.32		0.30		0.29		0.22	
Within-group	0.32	100%	0.29	96.67%	0.28	96.55%	0.21	95.45%
Between-group	0.00		0.01	3.3%	0.01	3.45%	0.01	4.54%

	IPTp coverage							
	2003		2008		2014		2022	
	Theil index	Contribution	Theil index	Contribution	Theil index	Contribution	Theil index	Contribution
Overall	57.15		1.32		0.79		0.33	
Within-group	57.14	99.98%	1.32	100%	0.79	100%	0.33	100%
Between-group	0.01	0.02%	0.00	0%	0.00	0%	0.00	0%

Source: Author computations

**Table 7:** Decomposition of the Theil indices by SES for treated net usage and IPTp coverage

	Overall within and between group inequalities by SES							
	ITN use in pregnancy							
	2003		2008		2014		2022	
	Theil index	Contribution	Theil index	Contribution	Theil index	Contribution	Theil index	Contribution
Overall	0.22		0.40		0.36		0.26	
Within-group	0.22	100%	0.39	98%	0.33	92%	0.22	85%
Between-group	0.00	0%	0.01	2.5%	0.03	8%	0.04	18%

	ITN use by Under 5 children							
	2003		2008		2014		2022	
	Theil index	Contribution	Theil index	Contribution	Theil index	Contribution	Theil index	Contribution
Overall	0.32		0.30		0.29		0.22	
Within-group	0.31	96.88%	0.29	96.67%	0.28	96.55%	0.20	90.9%
Between-group	0.01	3.13%	0.01	3.33%	0.02	6.7%	0.02	9.09%

	IPTp coverage							
	2003		2008		2014		2022	
	Theil index	Contribution	Theil index	Contribution	Theil index	Contribution	Theil index	Contribution
Overall	57.15		1.32		0.79		0.33	
Within-group	57.14	99.98%	1.31	99.24%	0.78	98.73%	0.32	97%
Between-group	0.01	0.02%	0.02	0.06%	0.01	1.27%	0.01	3%

Source: Author computations

A similar pattern holds for under-5 ITN use. The urban Theil index in 2008 is 0.50 (SE = 0.01, 95% CI 0.35–0.40), and the rural estimate is 0.23. These contribute to the total Theil index of 0.30 in Table 6 for that year, with within-group inequality accounting for 96.67% and between-group inequality for only 3.33%. Again, the SEs and CIs that apply to these decomposed components are those already reported in Table 4. For IPTp coverage, Table 5 reports a Theil index of 68.17 for urban women in 2003 (SE = 0.002, 95% CI 0.99–1.00) and 53.85 for rural women (no SE reported), which correspond to the overall inequality of 57.15 in Table 6. Here, 99.98% of the inequality is within-group and only 0.02% is between-group, with the precision already documented in Table 5. Table 7 (decomposition by SES), the total Theil index for ITN use among pregnant women in 2022 is 0.26. Table 4 shows subgroup indices: 0.12 for the poor (SE = 0.02, 95% CI 0.30–0.38), 0.28 for the middle, and 0.79 for the rich. The reported within-group contribution is 85%, and the between-group share is 18%. The standard errors for these subgroup estimates are reported in Table 4 and equally apply to the decomposition presented in Table 7. Likewise, for IPTp in 2003, Table 5 reports SES-specific indices: 53.06 for the poor (SE = 0.002, 95% CI 0.99–1.00), 63.00 for the middle, and 64.17 for the rich. These inputs yield a total Theil index of 57.15 in Table 7, where 99.98% is within-group and 0.02% between-group. Again, the SE and CI are already documented in Table 5.

## Discussion

This study aimed to examine both between- and within-socioeconomic group inequalities in the uptake of malaria prevention among pregnant women and children under five in Ghana between 2003 and 2022. Findings showed that ITN use increased modestly over the period, especially among under-five children (from 58.1% to 62.9%), with consistently higher uptake among rural and poorer households. In contrast, IPTp coverage rose sharply from 0.85% in 2003 to 59.4% in 2022 but remained pro-rich throughout. Most of the observed inequality stemmed from within-group variation rather than differences between socioeconomic or residential groups.

Despite nearly two decades of malaria control efforts, ITN use among children under five in Ghana increased by only 4.75 percentage points; an underwhelming gain with no statistical significance. This slow progress underscores persistent systemic barriers, as noted by Nuñez et al. [12] regional disparities in ITN access remain stark, often eroding national-level achievements. Without addressing structural bottlenecks such as unreliable supply chains, inconsistent funding, and weak community engagement, ITN interventions will continue to fall short of their potential [43–47]. Nonetheless, Ghana has made important strides by institutionalizing mass distribution campaigns and integrating ITN delivery through community-based health planning and services (CHPS), which have been shown to improve household access and usage in several districts [48]. Policy action must now build on these gains by prioritizing equitable distribution equitable distribution, sustained financing, and localized behaviour change strategies to accelerate and sustain gains.

Among pregnant women, ITN use followed a fluctuating pattern; declining in 2008 before rising again by 2022, though the overall change was not statistically significant. Previous studies by Kanmiki et al. [9] and Nlinwe et al*.* [5] suggest that this decline may be linked to supply chain disruptions and shifts in distribution strategies. Additionally, the transition from donor-driven to government-led distribution systems may have led to temporary disruptions [49–51]. Encouragingly, ITN use improved in later years likely due to Ghana’s sustained integration of ITN delivery into antenatal care clinics (ANC) as well as child welfare clinics (CWC), a strategy that has proven effective in reaching high-risk groups [12]. To consolidate and scale these gains, policy efforts should prioritize the scaling up of institutionalization of ANC- and CWC-based ITN delivery nationwide, while ensuring consistent funding, timely supply chain coordination, and monitoring to prevent future disruptions.

Of the three malaria prevention strategies, IPTp coverage saw the most pronounced and consistent rise over time. This upward trend reflects Ghana’s effective integration of IPTp into routine ANC; a strategy strongly endorsed by both national health authorities and global health partners. Embedding IPTp within the standard ANC package ensures timely administration, repeated contact opportunities, and continuous health education, all of which contribute to higher uptake [52–54]. Alonso et al*.* [55] affirm that such institutional integration is critical to broadening access and sustaining adherence. Complementary efforts, including training healthcare providers, ensuring SP availability, and promoting community awareness, have further reinforced coverage gains. The steady rise in IPTp uptake is a clear indicator of successful health systems strengthening and policy prioritization of maternal health. To sustain this momentum, continued investment in ANC platforms, supply chain reliability, and routine performance monitoring remains essential.

Findings also reveal significant socioeconomic and urban–rural disparities in ITN use and IPTp coverage. Among both pregnant women and children under five, ITN uptake was consistently higher in rural areas compared to urban settings**.** This rural advantage in usage likely reflects both targeted interventions and higher malaria burden in rural areas. As noted by Nlinwe et al*.* [5] and Kanmiki et al*.* [9] rural communities often benefit from mass ITN campaigns and community-based outreach, which are more logistically feasible and frequently prioritized in high-transmission zones. However, urban–rural gap indicates the need for tailored malaria control strategies in urban settings especially in densely populated areas where transmission risks can still be substantial [56]. Although overall malaria prevalence tends to be lower in urban areas, localized hotspots driven by systemic weaknesses such as inadequate housing, poor drainage, poor enforcement of environmental health regulations, and limited access to vector control services [56–59]. This calls for tailored interventions in urban settings, such as improved urban planning to reduce mosquito breeding sites, targeted distribution of ITNs, and increased public awareness campaigns. As recent studies have emphasized, one-size-fits-all approaches are insufficient; effective malaria control necessitates nuanced, context-compliant interventions responsive to the evolving epidemiology of malaria across rural and urban settings [56–59].

Socioeconomic disparities were also evident, with the highest ITN use among the poorest households and the lowest among the middle class. This pro-poor distribution pattern, observed in both children and pregnant women, aligns with findings by Were et al*.* [60] in Kenya, Bawuah and Ampaw, [61] in Ghana, Ojo et al*.* [62] in Nigeria, Haileselassie et al*. *[63] in Kenya, Wafula et al*.* [64] in sub-Saharan Africa, who noted similar trends. These studies suggest that ITN distribution programs often prioritize the most vulnerable populations, including the poorest households, to maximize the impact on malaria control. This targeted approach is crucial for ensuring that the most at-risk groups receive adequate protection.

However, the present study’s findings show increasing concentration indices for ITN use among pregnant women in 2022 and children under five in 2022 indicate widening inequalities over time. This suggests that while ITN distribution has reached the poorest, it has not sufficiently penetrated higher socioeconomic groups, a concern also raised by Nlinwe et al*.* [5]. The widening gap may be attributed to several factors, including differences in health-seeking behaviours, availability of alternative malaria prevention methods among wealthier groups, and potential barriers in accessing free or subsidized ITNs [65]. Wealthier households might prefer purchasing ITNs or using other preventive measures such as indoor residual spraying, aerosol insecticide sprays, and mosquito coils leading to lower uptake of distributed ITNs [47]. It is also plausible that wealthier people live in less malaria-prone areas compared to low SES people [61]. To close this equity gap, malaria control strategies should adopt a dual approach; strengthen free or subsidized ITN access and promote consistent use among low-income households, while also improving reach among higher-income groups who remain underrepresented in public ITN programmes. For these groups, this could involve distributing ITNs through private health providers, linking access to health insurance or work place schemes, and tailoring messaging to resonate with diverse health priorities.

For IPTp coverage, this study found a shift from minimal inequality in 2003 to a pro-rich distribution in subsequent years. This indicates that wealthier women are more likely to receive IPTp, primarily due to three interrelated factors; higher rates of ANC attendance, which increases opportunities to receive IPTp doses during scheduled visits [66]; fewer financial and logistical barriers, such as transport costs or long wait times, that often discourage consistent care-seeking among poorer women [67–69].; and greater awareness of IPTp benefits and guidelines due to higher educational attainment [67, 70, 71]. This pro-rich trend points towards the persistent inequities in maternal healthcare access. Addressing these gaps requires targeted measures to improve ANC uptake among lower-income women such as removing user fees [68, 72], expanding service delivery through mobile clinics [73]. and strengthening primary care infrastructure in underserved areas [74, 75]. Additionally, tailored community education and provider training can help ensure consistent IPTp delivery and uptake across all socioeconomic groups [68, 76].

Theil indices revealed that socioeconomic inequalities in ITN use and IPTp coverage were predominantly within-group rather than between-group, indicating that intra-group disparities (e.g., within urban or rural areas) are more significant. This pattern mirrors findings by Nuñez et al*.*, who emphasized the role of localized factors in shaping health inequalities [12]. Specifically, factors such as community health infrastructure, local health beliefs, and the effectiveness of local health programmes contribute significantly to the disparities observed within groups [77–80]. For instance, even within urban areas, neighbourhoods with better access to healthcare facilities and higher health literacy rates exhibit higher ITN use and IPTp coverage compared to less advantaged neighbourhoods. Moreover, the increase in between-group contributions over time, particularly for IPTp coverage, suggests growing disparities between different socioeconomic groups. This trend necessitates targeted interventions to address the barriers faced by poorer and less educated women, as highlighted by Hill et al*.* [68] and Roman et al*.* [52], who discussed similar challenges in their analysis of health intervention uptake in sub-Saharan Africa. The growing between-group disparities indicate that while overall access to interventions like IPTp may have improved, the benefits are not equitably distributed across different socioeconomic strata. Wealthier and more educated women are better positioned to take advantage of health services due to fewer financial constraints, better health awareness, and more frequent interactions with healthcare providers [81, 82].

The analysis of within-group inequalities also points to significant variations in ITN use and IPTp coverage based on localized socioeconomic factors. These disparities are often shaped by geographic barriers, with communities in remote areas facing limited availability of health services, longer travel distances to clinics, and weaker distribution infrastructure [83–85]. Even within rural settings, some areas benefit from better outreach, while others remain underserved. In urban areas, malaria prevention inequalities are strongly shaped by socioeconomic stratification [86]. Wealthier and more educated urban residents are more likely to access and utilize ITNs and IPTp [87, 88]. In contrast, poorer urban populations may face exclusion from public health interventions despite geographical access to facilities [89]. This calls for urban malaria strategies that explicitly address intra-urban disparities through tailored delivery models and targeted outreach to marginalized communities.

The increase in between-group contributions, particularly in the later years of the study, underscores the need for policies that not only increase overall coverage of ITN and IPTp but also specifically target the less privileged groups. This includes initiatives such as subsidized or free ITN distribution [47], mobile clinics providing IPTp in underserved areas [68], and educational campaigns tailored to lower-income and less-educated populations to raise awareness about the importance of these interventions [90]. As suggested by Sacca et al*.* [91], addressing these barriers involves understanding and mitigating the specific challenges faced by these groups, such as transportation costs, opportunity costs of attending health facilities, and cultural beliefs that may hinder the uptake of health interventions. Furthermore, the decomposition of Theil indices by residence shows that the socioeconomic inequalities in ITN use and IPTp coverage are explained mostly by variations within urban or rural settings rather than between these settings. This highlights the importance of intra-group factors, such as local health service delivery efficiency, community engagement in health programs, and socioeconomic dynamics within communities. It also suggests that policies and interventions need to be finely tuned to address these within-group disparities to be more effective. The reduction in within-group inequalities over time, coupled with the rise in between-group inequalities, could reflect the homogenization of certain community groups while accentuating the divide between different socioeconomic groups. This indicates a trend where local interventions might be improving overall but are not sufficient to bridge the gap between the rich and the poor or the educated and the less educated. Therefore, it is crucial to implement strategies that specifically address these growing between-group inequalities to ensure more equitable health outcomes.

### Strengths and limitations

This study leverages four rounds of nationally representative DHS data to examine both between-group and within-group socioeconomic inequalities in ITN and IPTp coverage among pregnant women and children in Ghana. Its other strengths include the use of robust inequality decomposition methods and a unique focus on hidden disparities within subpopulations, which are often overlooked in malaria intervention research. However, limitations include the reliance on cross-sectional, self-reported data and the constrained scope of explanatory variables. Despite these limitations, the findings offer policy-relevant insights that can support more equitable malaria programming aligned with national elimination goals.

### Policy implications

This study’ findings have several policy implications. First, the modest gains in ITN use among children under five and the inconsistent trends among pregnant women call for stronger programme continuity. This includes sustained investment in the ITN supply chain, improved last-mile delivery systems, and real-time stock monitoring to avoid coverage gaps [12]. Second, pronounced urban–rural and wealth-related disparities in both ITN and IPTp use highlight the need for context-sensitive interventions. These may involve targeted distribution strategies in underserved rural areas, integration of ITNs into private-sector urban health networks, and customized outreach to marginalized urban populations, as suggested by Nuñez et al*.* [12] and Budu et al*.* [14]. Third, the pro-rich distribution of IPTp coverage reinforces the importance of removing barriers to antenatal care. Key strategies include fee waivers, mobile ANC clinics, and tailored health education that empowers lower-income women to initiate and complete IPTp regimen [9]. With Ghana’s 2024–2028 National Malaria Elimination Strategic Plan now underway, the call for bold, targeted action is urgent [91]. The NMESP sets ambitious goals to “reduce malaria mortality by 90%,” “cut case incidence by 50%,” and “eliminate malaria in 21 low-burden districts by 2028”. Achieving these targets demands a laser focus on equity, especially for pregnant women and children under five, by scaling up context-specific strategies that close persistent socioeconomic and geographic gaps in ITN use and IPTp uptake.

## Conclusion

Despite gains in malaria prevention coverage in Ghana, significant socioeconomic and geographic inequalities persist. Results highlight that most disparities are driven by within-group differences, with growing between-group inequality, especially in IPTp uptake. Addressing these inequities requires a dual approach: sustaining universal access and targeting underserved subgroups through context-specific interventions. Tailored strategies that address local barriers are essential to ensure equitable health outcomes and achieve Ghana’s malaria elimination and UHC goals.

## Data Availability

No datasets were generated or analysed during the current study.
